# Clinical Benefit of Cancer Philosophy Clinic for Cancer Patients Using EQ-5D-5L Scores

**DOI:** 10.14789/jmj.JMJ19-Re04

**Published:** 2023-03-29

**Authors:** MAYO NUNOKAWA, EIICHI INADA, OKIO HINO

**Affiliations:** 1 Department of Anesthesiology and Pain Medicine, Juntendo University School of Medicine, Tokyo, Japan; 1 Department of Anesthesiology and Pain Medicine, Juntendo University School of Medicine, Tokyo, Japan; 2 Department of Pathology and Oncology, Juntendo University School of Medicine, Tokyo, Japan; 2 Department of Pathology and Oncology, Juntendo University School of Medicine, Tokyo, Japan

**Keywords:** cancer philosophy clinic, cancer patients, EQ-5D-5L

## Abstract

**Objective:**

This study aims to understand the role of Cancer Philosophy Clinic activities among participants and whether participation is correlated to increase in QOL.

**Materials and Methods:**

Among the 150 Cancer Philosophy Clinics, questionnaire surveys were distributed at 28 locations that consented to participating in the study. The data was analyzed based on the respondent’s situation and health related Quality of Life (QOL) prior to and after participating in Cancer Philosophy Clinic using the EQ-5D-5L questionnaire (Japanese version) regarding health related QOL prior to and after participating in Cancer Philosophy Clinic.

**Results:**

There were more female participants than male participants; 224 and 76 respectively. 46.5%, or approximately half of all participants in the Cancer Philosophy Clinic were “cancer patients,” followed by 17.2% who were “family members of cancer patients,” 16.6% who were “not suffering from any diseases,” 11.4% who were “suffering from diseases other than cancer” and 3.2% who were classified as “other,” who were bereaved family members. 51.7% were “currently receiving treatment, “32.1% were “receiving follow-up medical care, “and 15.3% were “survivors.” There was 1 participant who commented, “refusing treatment.” Based on an evaluation of QOL using EQ-5D-5L of 184 participants who were participating in the Cancer Philosophy Clinic, an increase in overall average index value from 0.827 to 0.867 was observed after participation compared to prior participation. In particular, there was a significant improvement in “pain/discomfort,” “anxiety/depression.

**Conclusions:**

Cancer Philosophy clinic has been found important role in encouraging existing shift.

## Introduction

The population in Japan is aging significantly. As aging progresses, there is greater concern for developing various diseases. Aging is one factor in developing cancer, dementia, cerebrovascular disease, and cardiovascular disease. According to a study by the Ministry of Health, Labour, and Welfare^1)^, a breakdown of inpatients and outpatients in 2014 by disease and related health problems found the greatest number of inpatients suffering from “V mental and mobile disability,” (265.5 thousand) “XI cardiovascular disease,” (240.1 thousand) and “II neoplasm” (144.9 thousand). The greatest number of outpatients was suffering from “XI digestive disease,” (1.31 million), “XI cardiovascular disease,” (933 thousand), and “XIII musculoskeletal and connective tissue disease,” (877.8 thousand. 1) malignant growths, or cancer is the third most common disease among inpatients. Currently, cancer rates are rising and 1 in 2 patients are said to be cancer patients. Cancer is the most common cause of death in Japan.

Cancer patients suffer not only from physical pain but also from psychological pain, mental illness, and spiritual pain^2)^. There are a variety of cases especially among cancer patients varying from those who respond well to cancer treatment, those whose condition remains unchanged, and those whose condition worsens; the cancer site, progression, medicine and treatment methods. QOL (Quality of Life) declines frequently especially in cases of progressive cancer and mental and psychological pain. It is clear that there is a tremendous impact on the QOL of patients. Psychological pain excluding pathological mental condition is described as spiritual pain. Many patients suffer from spiritual pain and experience anxiety, denial and despair toward themselves, and feel a tremendous sense of isolation and anger with no outlet.

Cancer Philosophy is a combination of “Political Philosophy,” by Shigeru Nanbara（First President of University of Tokyo after the war, 1889-1974）and “Cancer Studies,” by Tomizo Yoshida (General Manager of Cancer Research Institute, Professor of Tokyo University, General Manager of Sasaki Laboratory, 1903-1973） proposed by Okio Hino. (Professor of Pathology and Oncology Juntendo University)^3)^.

Cancer Philosophy Clinic was started as a series of five trials at Juntendo University Hospitals as an interactive outpatient service. There were many cancer patients and patients suffering from mental illness who wished to confide in someone their anxieties and thoughts. There were many participants not only from Tokyo but from other prefectures.

Cancer Philosophy Clinic is a place where patients suffering from anxiety, anger, and isolation, and those in any situation are accepted and supported. Currently there are 150 Cancer Philosophy Clinic locations nationwide, each with a representative. The number of times Cancer Philosophy Clinic is held and capacity vary from location to location. A small group of participants gather around a table to speak and listen over tea. Listeners do not deny what the speaker says, but listen, empathize and provide their opinions and advice based on their personal experiences. For those who cannot speak, participants may quietly support or speak to the person. If a coordinator facilitates, they summarize and present the opinions of participants at each table and sometimes share what was discussed with other tables. Cancer Philosophy Clinic activities are gradually increasing. Needless to say, there is an increasing demand. However, there has been little research on the needs and the role of Cancer Philosophy Clinic. There has been no research done on the participants attributes and the influence Cancer Philosophy Clinic has on increases in health related QOL.

## Objective

This study aims to understand the role of Cancer Philosophy Clinic activities among participants and whether participation is correlated to increase in QOL.

## Methods

Among the 150 Cancer Philosophy Clinics, questionnaire surveys were distributed at 28 locations that consented to participating in the study. The data was analyzed based on the respondent’s situation and health related Quality of Life (QOL) prior to and after participating in Cancer Philosophy Clinic using a EQ-5D-5L questionnaire (Japanese version) regarding health related QOL prior to and after participating in Cancer Philosophy Clinic.

### Attribute

1) Age and Gender

2) Presence of disease

3) Situation of Treatment (for cancer patients and those who responded as suffering from diseases other than cancer)

4) Participation in Cancer Philosophy Clinic

### Index Value of QOL

Health related QOL prior to and after participation in Cancer Philosophy Clinic was measured using EQ-5D-5L (Japanese version).

EQ-5D-5L is a standardized measure of health status which scores “mobility,” “self-care,” “usual activities,” “pain/discomfort,” “anxiety/depression” on a 5-level system.

### Period

3 months from June 2018 to August 2018.

## Results

### Attribute

#### 1)Age and Gender (Figure 1)

There were more female participants than male participants; 224 and 76 respectively. The greatest number of participants was in their fifties, accounting for 32.4% (99 participants) of total responses, followed by 24.99% (76 participants) in their sixties, 24.5% (75 participants) in their seventies, 12.4% (38 participants) in their forties, 3.2% (10 participants) in their twenties and 2.2% (7 participants) in their thirties, with no participants under the age of 20.

**Figure 1:**
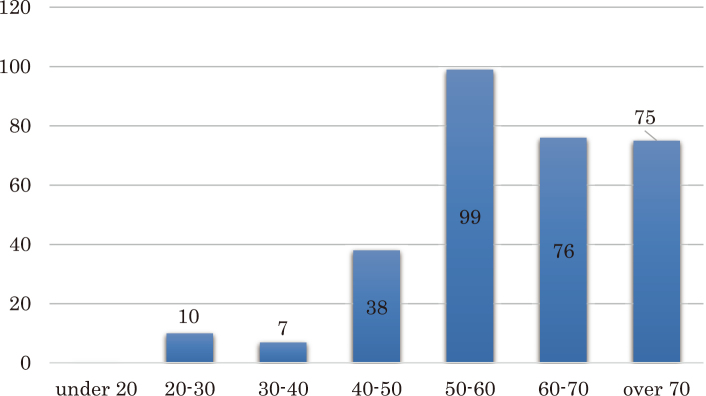
Age The greatest number of participants was in their fifties, accounting for 32.4% (99 participants) of total responses, followed by 24.99% (76 participants) in their sixties, 24.5% (75 participants) in their seventies, 12.4% (38 participants) in their forties, 3.2% (10 participants) in their twenties and 2.2% (7 participants) in their thirties, with no participants under the age of 20.

#### 2)Presence of disease (Figure 2)

46.5% (143 participants), or approximately half of all participants in the Cancer Philosophy Clinic were “cancer patients,” followed by 17.2% (53 participants) who were “family members of cancer patients,” 16.6% (51 participants) who were “not suffering from any diseases,” 11.4% (35 participants) who were “suffering from diseases other than cancer” and 3.2% (10 participants) who were classified as “other,” who were bereaved family members.

**Figure 2:**
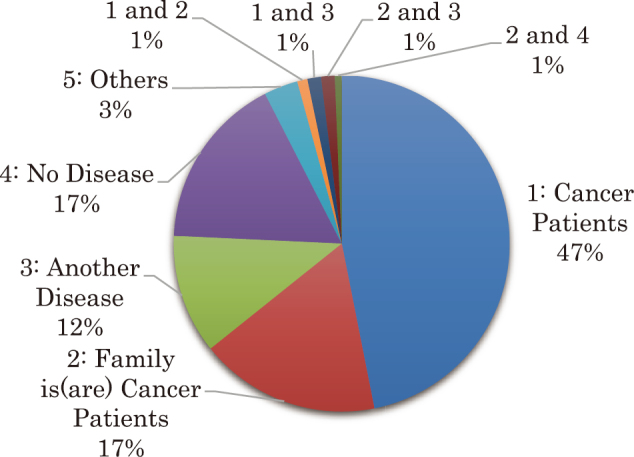
The Presence of Cancer 46.5% (143 participants), or approximately half of all participants in the Cancer Philosophy Clinic were “cancer patients,” followed by 17.2% (53 participants) who were “family members of cancer patients,” 16.6% (51 participants) who were “not suffering from any diseases,” 11.4% (35 participants) who were “suffering from diseases other than cancer” and 3.2% (10 participants) who were classified as “other,” who were bereaved family members.

#### 3)Situation of Treatment (Figure 3)

Among the 143 respondents who identified as “cancer patients” or “family members of cancer patients,” 51.7% (74 participants) were “currently receiving treatment, “32.1% (46 participants) were “receiving follow-up medical care, “and 15.3% (22 participants) were “survivors.” There was 1 participant who commented, “refusing treatment.”

**Figure 3:**
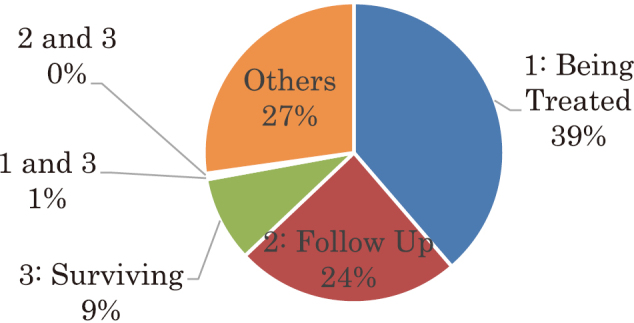
Situation of Treatment Among the 143 respondents who identified as “cancer patients” or “family members of cancer patients,” 51.7% (74 participants) were “currently receiving treatment, “32.1% (46 participants) were “receiving follow-up medical care, “and 15.3% (22 participants) were “survivors.” There was 1 participant who commented, “refusing treatment.”

#### 4)Participation in Cancer Philosophy Clinic (Figure 4)

21.9% (67 participants) “participated for the first time,” 9.1% (28 participants) “participated for the second time,” and 68.7% (210 participants) “participated three or more times,” indicating that there were many participants who had participated multiple times.

Based on an evaluation of QOL using EQ-5D-5L (Japanese edition) of 184 participants who were “cancer patients,” “suffering from diseases other than cancer,” or both participating in the Cancer Philosophy Clinic, an increase in overall average index value from 0.827 to 0.867 was observed after participation compared to prior participation. Among the 187 participants, change in QOL was observed in 47 participants, of which an increase in QOL was observed in 39 participants, and a decrease in QOL in 8 participants. In the details, 46 participants of the 47 participants changed and good improvement was confirmed. In particular, there was a significant improvement in “pain/discomfort,” “anxiety/depression” (Figure 5).

**Figure 4:**
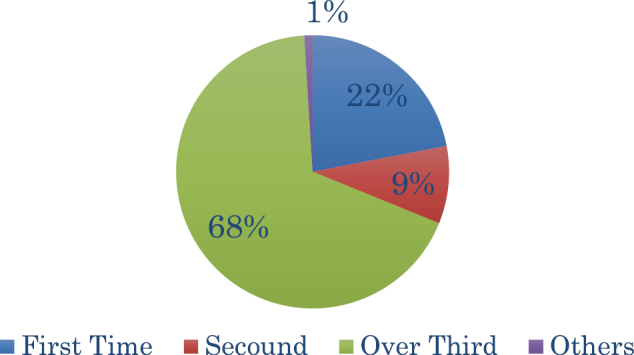
Number of Participated in Cancer Philosophy Clinic 21.9% (67 participants) “participated for the first time,” 9.1% (28 participants) “participated for the second time,” and 68.7% (210 participants) “participated three or more times,” indicating that there were many participants who had participated multiple times.

**Figure 5:**
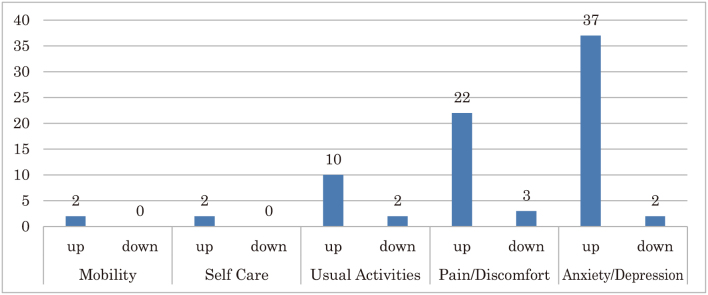
Changes after Participation 5 Scores of 47 Participants 46 participants of the 47 participants changed and good improvement was confirmed each 5 scores.

## Discussion

### Attribute

Participants in Cancer Philosophy Clinic in their fifties account for 32.4% of all participants, and 81.8% of all participants including those in their sixties and seventies. According to “Cancer registration and statistics” data from the National Cancer Center Information Service in 2014, cancer rates increase past the age of 50. As such, more people participate in Cancer Philosophy Clinic. In addition, women are more interdependent and tend to participate more actively in their communities than men^4)^.

Approximately half of all participants are “cancer patients” and suffer from mental and psychological distress from being diagnosed with cancer. It is clear that emotional support beyond physical treatment and support from doctors at hospitals is necessary. Nowadays, “cancer” does not automatically equate to “death,” but is rather considered a treatable disease. Nonetheless, one may become more aware of “death” in many cases. Depending on the progression of an illness, treatment may be difficult in some cases. It is known that there is a five-stage model in accepting death and dying^5)^.

In the first stage of denial and isolation, a person who is given a diagnosis of terminal illness is in shock and disbelief, and often isolates oneself to avoid the situation. In the second stage of anger, they may start to ask, “Why me?” and experience strong resistance. In the third stage of bargaining, they accept that they are dying, but try to bargain to avoid death from a desire to live a little longer. In the fourth stage of depression, they experience sadness, despair, and at times a feeling emptiness and accept that death is unavoidable. In the fifth stage of acceptance, they overcome the denial, avoidance, and feeing of emptiness experienced in earlier stages. It is a stage of peaceful resolution that death will occur. In this stage, a person quietly reflects upon their life and regains emotional peace and calm. At Cancer Philosophy Clinic, participants in various stages can gather and share their thoughts over tea with those in similar situations.

17.2% of participants were “family members of cancer patients.” When a family member suffers from cancer, there is a deep sadness and anxiety felt as if it were one’s personal situation. Many of these participants express concern and anxiety because they are unsure how to interact with the family member. At Cancer Philosophy Clinic, participants can listen to candid opinions of cancer patients and those suffering from other diseases that are difficult to treat. It is a space where they can confide in others their concern and anxiety. It is thought that having someone listen leads to mental stability. The sadness and loneliness experienced by the loss of family, friends, and close ones is immeasurable. The high number of bereaved family members indicates that Cancer Philosophy Clinic helps ease sadness and anger and serves the purpose of providing grief care.

Among the 307 respondents, 143 participants were “cancer patients,” of which 51.7% were “currently receiving treatment.” It is thought that participation leads to a broader perspective regarding the availability of alternative treatment options or perspectives, as well as whether the current treatment and its direction are appropriate. At Cancer Philosophy Clinic, there are many participants who question the direction of their treatment and are considering seeking a second opinion. Cancer Philosophy Clinic is a forum to gather valuable information for those currently receiving treatment.

68.7% of participants have participated in Cancer Philosophy Clinic three or more times indicated a high number of participants who have participated multiple times. It is thought that visiting Cancer Philosophy Clinic several times and talking to those in similar situations leads to physical and mental stability. In addition, for those who are in poor condition and unable to leave the house, services such as online, letters and messages need to be enhanced.

### Health related QOL

The QOL of 184 “cancer patients,” “those suffering from diseases other than cancer,” or both was scored using EQ-5D-5L (Japanese version). EQ- 5D was developed by EuroQol, a research group established in 1978. EQ-5D-5L is a new version that was later developed which can be used as a quantitative measure of health related QOL. The system is comprised of five dimensions, which the patient is asked to rate his or her health on five levels. A conversion table is used to describe the patient’s state of health^6)^.

An increase in overall average index value from 0.827 to 0.867 was observed after participation compared to prior participation. Change in QOL was observed in 47 participants, of which an increase in QOL was observed in 39 participants, and a decrease in QOL in 8 participants.

The respondents of this survey who were able to participate in Cancer Philosophy Clinic, therefore they were under treatment but had relatively preserved or relaxed ADL. As a result, index value averaged from 0.827 before participation to 0.867 after participation. Another study found that the epidemiological survey of nonspecific neck pain in the general population using EQ-5D, it can be said that the QOL of the group “with neck pain averaged 0.828” and that of the group “without neck pain averaged 0.919^7)^”. In another survey, the QOL assessment for postoperative patients of colorectal cancer, the score was “0.867^8)^”, and a large-scale survey on the impact of chronic pain on QOL, “0.71 with chronic pain” and “0.89 without chronic pain” were found to preserve quality of life better than those with chronic pain^9)^.

Half of all participants of Cancer Philosophy Clinic are “cancer patients,” of which half are “currently receiving treatment.” Normally, it can be inferred that cancer physically and mentally debilitates patients, and thus a decrease in QOL score could be predicted.

Changes in scores of the five dimensions: “mobility,” “pain/discomfort,” “self-care,” “usual activities,” and “anxiety/depression” were observed.

As a result, there were a significant number of participants who saw an improvement in “anxiety/depression.” We found that participating in Cancer Philosophy Clinic leads to easing anxiety and depression. It could be said that Cancer Philosophy Clinic greatly contributes to easing mental and emotional distress.

On the other hand, a study of the 8 participants whose QOL declined indicated that there were many cases where “usual activities,” “pain/discomfort” worsened. It is thought that the increase in pain and discomfort caused by diseases limits the patient’s daily activities, which leads to increased anxiety.

## Conclusions

For those who have been diagnosed with cancer or other diseases, being able to recognize an improvement in QOL, with improvement in “anxiety/depression” in particular is an affirmation of accepting reality and modifying one’s behavior in a positive manner. It is apparent that Cancer Philosophy clinic has an important role in encouraging existing shift^10)^ positive manner.

## Limitation

This survey was based on feedback from 28 out of 150 Cancer Philosophy Clinic, thus it is unclear whether the quality of all Cancer Philosophy Clinic has been assured or whether all clinics will show the same usefulness. In addition, in this study, only EQ-5D-5L was used to evaluate the clinical benefit.

## Funding

The authors declare that they have no conflict of interest.

## Author contributions

MN and OH conceived the idea of the study. EI and OH developed the statistical analysis plan and conducted statistical analyses. OH supervised the conduct of this study. All authors reviewed the manuscript draft and revised it critically on intellectual content. All authors approved the final version of the manuscript to be published.

## Conflicts of interest statement

The authors declare that they have no conflict of interest.
